# Brain-related proteins as serum biomarkers of acute, subconcussive blast overpressure exposure: A cohort study of military personnel

**DOI:** 10.1371/journal.pone.0221036

**Published:** 2019-08-13

**Authors:** Angela M. Boutté, Bharani Thangavelu, Christina R. LaValle, Jeffrey Nemes, Janice Gilsdorf, Deborah A. Shear, Gary H. Kamimori

**Affiliations:** 1 Brain Trauma Neuroprotection Branch, Center for Military Psychiatry and Neuroscience, Walter Reed Army Institute of Research, Silver Spring, Maryland, United States of America; 2 Blast Induced Neurotrauma Branch, Center for Military Psychiatry and Neuroscience, Walter Reed Army Institute of Research, Silver Spring, Maryland, United States of America; University of Florida, UNITED STATES

## Abstract

Repeated exposure to blast overpressure remains a major cause of adverse health for military personnel who, as a consequence, are at a higher risk for neurodegenerative disease and suicide. Acute, early tracking of blast related effects holds the promise of rapid health assessment prior to onset of chronic problems. Current techniques used to determine blast-related effects rely upon reporting of symptomology similar to that of concussion and neurocognitive assessment relevant to operational decrement. Here, we describe the results of a cross sectional study with pared observations. The concentration of multiple TBI-related proteins was tested in serum collected within one hour of blast exposure as a quantitative and minimally invasive strategy to augment assessment of blast-exposure effects that are associated with concussion-like symptomology and reaction time decrements. We determined that median simple reaction time (SRT) was slowed in accordance with serum Nf-L, tau, Aβ-40, and Aβ-42 elevation after overpressure exposure. In contrast, median levels of serum GFAP decreased. Individual, inter-subject analysis revealed positive correlations between changes in Nf-L and GFAP, and in Aβ-40 compared to Aβ-42. The change in Nf-L was negatively associated with tau, Aβ-40, and Aβ-42. Participants reported experiencing headaches, dizziness and taking longer to think. Dizziness was associated with reaction time decrements, GFAP or NfL suppression, as well as Aβ peptide elevation. UCH-L1 elevation had a weak association with mTBI/concussion history. Multiplexed serum biomarker quantitation, coupled with reaction time assessment and symptomology determined before and after blast exposure, may serve as a platform for tracking adverse effects in the absence of a head wound or diagnosed concussion. We propose further evaluation of serum biomarkers, which are often associated with TBI, in the context of acute operational blast exposures.

## Introduction

Overpressure (OP) is defined as the pressure caused by a shock wave that exceeds normal atmospheric pressure. OP exposure (Exp) may be caused by a variety of explosive devices or charges, as well as munitions. A subset of military personnel, “breachers”, use a tactical technique to force entry into a closed area and within structures experience and are regularly exposure to repeated exposure. Low levels of exposure are linked to acute reduction in operational performance indicated, in part, by a decrement in reaction time (RT) [1]. Repetitive exposure has been linked to a complex array of symptoms including headaches, tinnitus, fatigue, and dizziness. This symptom complex has been termed “breacher’s brain” [2–4]. Symptoms are similar to those observed among persons who have a clinically diagnosed mild traumatic brain injury (mTBI) or concussion, one of the most common injuries sustained by military personnel, particularly those who engage in training and combat roles (http://dvbic.dcoe.mil/tbi-military). These effects are often transient, underreported, and challenging to identify due to symptom variability; which makes classification of an objective “injury response” difficult to achieve.

Assessment of exposure mediated effects as they relate to performance, resilience, or mTBI, are often achieved through neurocognitive testing. The Defense Automated Neurobehavioral Assessment (DANA) is a field deployable neurocognitive and psychological assessment tool developed and extensively tested by the Department of Defense [5]. The DANA was commissioned to assist with detecting performance change over a variety of issues, such as concussion, occurring in combat deployment settings. The DANA has been tested in several operational environments and being used in research contexts [1, 6]. More recently, the use of blood based biomarkers have been suggested to further augment stratification and, potentially, health status relevant directly to exposure.

Central nervous system-enriched proteins, such as neurofilament light chain (Nf-L), tau, and glial fibrillary protein (GFAP) have been used as objective measurements to identify TBI [7, 8]. Similarly, ubiquitin carboxy-terminal hydrolase L1 (UCH-L1) and amyloid precursor protein (APP), the precursor of amyloid beta (Aβ) peptides, have been identified in peripheral blood collected weeks-months after exposure [9]. These biomarkers have been robust identifiers within the context of chronic paradigms relevant to overpressure as well as TBI. Yet, despite the high incidence of exposure and the likelihood of symptomology similar to mTBI, an objective and quantifiable evidence of an “injury effect” per biomarker assessment during an acute, or near immediate time frame, remains elusive. Therefore, this preliminary study was conducted to determine the changes in reaction time, self-reported symptoms, and quantitation of TBI-associated serum biomarkers among military personnel exposed to overpressure exposure caused by blast. Early, sensitive quantitation of exposure-mediated peripheral biomarkers may be capable of identifying biological effects of overpressure and augment in-field care, even in the absence of a fully diagnosed concussion or visible traumatic brain injury.

## Materials and methods

### Study participants

Active duty United States Army personnel (n = 29) within a single site, Fort Leonard Wood, MO engaged in a two-week breacher training course. Heavy wall breaching exercises occurred within one training day during which neurocognitive testing, blood sampling, and symptomology assessments were conducted.

### Overpressure measurements

All participants were exposed to two overpressure events (back-to-back heavy wall breaches to defeat concrete walls) in a single training session. Exposure levels were measured as psi (pound per square inch) using the B3-H pressure sensor mounted on the left shoulder of each participant to approximate incident pressure. The B3-H is a small, lightweight, accurate, disposable, and off the shelf device that records and collects data peak pressure, acceleration (rate at which speed changes) and impulse (time exposed to certain levels of overpressure exposure). Peak pressure (psi) and impulse (psi X milliseconds [ms]) are displayed for each incident and as cumulative values for the training session. Participants are in a static position during exposure. Therefore, acceleration does not occur and does not meet the threshold to be automatically recorded by the B3-H sensor.

### Assessment of neurocognitive performance

The Defense Automatized Neurocognitive Assessment (DANA) tool was administered prior to (pre: -8h) and after (post: +1h) Exp in accordance with blood-draw time. The DANA consists of three subtasks conducted with a hand-held device and monitor screen. (1) Simple reaction time (SRT) measures pure reaction time. The participant was required to tap on the location of the yellow asterisk symbol as quickly as possible each time it appeared; (2) Procedural reaction time (PRT) is a choice reaction time that measures accuracy, reaction time, and impulsivity. The screen displays one of four numbers for 3 seconds (sec). The participant was required to press a left button (“2” or “3”) or right button (“4” or “5”). This choice reaction time task targets simple executive functioning and working memory; and (3) Go-No-Go (GNG) is a forced choice reaction-time task. A picture of a house is presented on the screen. Either a “friend” (green) or “foe” (white) appeared in a window. The respondent must push a “fire” button only when a “foe” appears. The choice reaction time measures sustained attention and impulsivity. The test quantifies speed and accuracy of target omissions and commissions.

### Symptom and TBI/concussion history reporting

Participants completed a 32-item, paper-and-pencil health symptom inventory before (pre-) and after (post-) Exp, in conjunction with each blood draw. The symptoms on the inventory are similar to that of the Rivermead instrument [10, 11], but with additional items and responses relevant to the breaching exercise context rather than exclusively to concussion [12]. Participants were instructed to use a 5-point Likert scale (0 “not experienced at all,” 1 “no more of a problem than before training,” 2 “mild problem–present but don’t really notice and doesn’t concern me,” 3 “moderate problem–I can continue what I am doing but I notice the problem,” 4 “severe problem–constantly present, feels like it could affect my performance”). Participants noted prior history of concussion or mTBI which is reported as a binary metric (No = 0, Yes = 1). Clinical data was not available.

### Serum preparation and quantitative biomarker measurements

Venous blood was collected directly into BD Vacutainer SST Serum Separation Tubes (Fisher Scientific, Waltham, MA) and processed within 30 minutes according to the manufacturer’s instructions. Samples were centrifuged at 1,000 x g for 10 minutes, at room temperature. Samples were stored in 1mL aliquots, supplemented with HALT protease/phosphatase inhibitors, and then stored at -80 C until use. GFAP, UCH-L1, Nf-L, tau, Aβ-40, and Aβ-42 were measured using digital immunoassays performed using the Simoa HD-1 according to manufacturer’s instructions (Quanterix Corporation, Lexington, MA). All assays were performed based on manufacturer’s recommendations. Briefly, serum was thawed on ice then centrifuged at 10,200 x g for 10 minutes at 4° C. Thereafter, 120μL of serum supernatant was directly loaded onto a 96 well plate and diluted 1/4 during the assay. Curve fitting analysis was conducted using pre-set programs designed by the manufacturer.

### Data management and statistical analysis

The full dataset containing age (years), sampling time (hours), peak pressure (psi and kPa), impulse (psi X ms, DANA values (ms), biomarker concentrations [pg/mL], and dichotomized symptomology or mTBI/concussion history for each participant is shown (S1 Table). Non-dichotomized, Likert scale symptomology reporting is provided (S2 Table). All data was analyzed using Prism version 7 (GraphPad, La Jolla, CA). Biomarker concentrations [pg/mL] were compared using the Wilcoxon signed rank test, * p ≤ 0.05. Data is shown as the median concentration [pg/mL] +/- IQR. Symptomology was transformed into binary variables (No change or a decrease = 0 or “-”; an increase = 1, or “+”). Biomarker and DANA values were transformed into a delta (d = post-Exp minus pre-Exp) and outliers were removed (ROUT = 1%) prior to correlative analysis using 1-tailed, Spearman rank correlation coefficient, * p ≤ 0.05, or to comparisons against dichotomized (—, no change or decrease vs. +, increased) symptom data. Distribution of delta DANA or delta biomarker values were tested for normality using the D‘Agostino & Pearson test prior to comparison using 1-tailed Mann-Whitney U-Test or Welch’s t-Test as appropriate, * p ≤ 0.05, and are displayed as the median +/- 5–95%-ile range. Delta DANA or biomarker values compared to self-reported symptomology or to mTBI/concussion history are displayed (S3 Table).

## Results

Participants (n = 29) within this study (Table 1) were males aged 21–43 (mean: 29.5 yrs.), with variable duration of service (mean +/ SD: 8.5+/-4.6 yrs.; range: 2–20 yrs.). Acute blood sampling occurred within one hour following Exp (mean: 0.99h, range: 0.52–1.68h). The levels (mean +/-SD) of peak pressure (4.35+/-0.49 psi or 30.0+/-3.37 kPa / incident); cumulative peak pressure (8.71+/- psi or 60.0+/-6.83 kPa / session); impulse (11.7+/-1.13 psi X ms / incident); and cumulative impulse (23.5+/-2.26 psi X ms/session) derived from B3-H sensors mounted on each participant’s left shoulder are indicated. Participants experienced symptomology similar to concussion (Table 2). Headaches (15/29, 52%) and taking longer to think (12/29, 41%) were the most frequently reported, followed by dizziness (9/29, 31%), slowed thinking (8/29, 29%), and poor concentration (8/29, 28%).

**Table 1:** Demographic characteristics of study participants and biosample collection timelines.

**Number of Subjects (n)**	29	
**Age (years)**		
Mean (SD)	29 (5.1)	
Range [Min-Max]	21–43	
**Gender, No. (%)**		
Male	29 (100%)	
Female	0	
**Duration of Service**		
Mean (SD)	8.5(4.6)	
Range [Min-Max]	2–20	
**Post Exposure Sample Collection Time**	**Hours (h)**	
Mean (SD)	0.99(0.29)	
Range [Min-Max]	0.52–1.68	
**Peak Pressure**	**psi**	**kPa**
Per Incident Mean (SD)	4.35 (0.49)	30.0 (3.37)
Cumulative Mean (SD)	8.71 (0.99)	60.0 (6.83)
**Impulse**	**psi x time (milliseconds)**	
Per Incident Mean (SD)	11.7 (1.13)	
Cumulative Mean (SD)	23.5 (2.26)	

Participant age, gender, duration of service are shown. The post-Exp time-point of peripheral blood collection, DANA neurocognitive testing, and Exp levels of peak pressure (psi and kPa) as well as impulse (psi X time) derived from left shoulder B3-H sensors are displayed.

**Table 2:** Symptoms reported after overpressure exposure.

Symptom	Increase	Decrease or No Change	% Reporting Increase
Headaches	15	14	52
Taking longer to think	12	17	41
Feelings of dizziness	9	20	31
Slowed thinking	8	19	30
Poor concentration	8	21	28
Ringing in ears	5	23	18
Feeling anxious or tense	5	24	17
Blurred vision	5	24	17
Easily upset by loud noise	4	25	14
Being irritable or easily angered	4	25	14

The table indicating the number of participants and self-reported symptoms is shown. The top ten of 32 symptoms are shown with the number of participants reporting an increase, a decrease or no change. The percent (%) of participants who reported an increase is indicated.

DANA administration and serum biomarker testing revealed several changes relevant to OP (Table 3). Median SRT increased by 30.5 ms from pre-Exp (median: 269.0 ms, IQR: 256.6–305.7 ms) to post-Exp (median: 299.9 ms, IQR: 273.5–330.6 ms, p = 0.048). There was no difference in PRT (pre -median: 636.9 ms, IQR: 592.9–672.1 ms; post—median: 633.7 ms, IQR: 601.5–698.7 ms, 0.398) or GNG (pre—median: 604.7 ms, IQR: 554.0–655.7 ms, NS; post—median: 597.6 ms, IQR: 560.1–668.4 ms, 0.475). Serum GFAP concentrations fell marginally after Exp (pre–median: 59.2, IQR: 45.3–70.2; post–median: 52.1, IQR: 44.0–70.2, p = 0.051). There was no change in UCH-L1 (pre–median: 7.83, IQR: 2.53–22.1; post–median: 7.07, IQR: 3.00–20.5, 0.978). In contrast, Nf-L (pre–median: 4.90, IQR: 3.77–6.71; post–median: 5.23, IQR: 3.73–7.58, p = 0.035) and tau nearly doubled although it did not meet the statistical threshold (pre—median: 0.074, IQR: 0–0.22; post–median: 0.122, IQR: 0–0.23, p = 0.090). Aβ-40 displayed a considerable upward trend (pre–median: 126.0, IQR: 92.1–160.0; post–median: 140.0, IQR: 104.5–164.5, 0.151) and Aβ-42 levels higher (pre–median: 5.09, IQR: 2.47–6.55; post–median: 5.19, IQR: 3.80–7.01, p = 0.046).

**Table 3:** DANA and biomarker concentrations before and after overpressure exposure.

**DANA (milliseconds)**	**Pre-Exp**		**Post-Exp**		**% Change**	**p-value**
	Median	IQR	Median	IQR		
**SRT**	269.0	256.6–305.7	299.9	273.5–330.6	11.5	0.048*
**PRT**	636.9	592.9–672.1	633.7	601.5–698.7	-0.50	0.398
**GNG**	604.7	554.0–655.7	597.6	560.1–668.4	-1.17	0.475
**Serum Biomarker (pg/mL)**	**Pre-Exp**		**Post-Exp**		**% Change**	**p-value**
	Median	IQR	Median	IQR		
**GFAP**	59.2	45.3–70.2	52.1	44.0–70.2	-12.0	0.051
**UCH-L1**	7.83	2.53–22.1	7.07	3.00–20.5	-9.71	0.978
**Nf-L**	4.90	3.77–6.71	5.23	3.73–7.58	6.73	0.035*
**Tau**	0.07	0–0.22	0.122	0–0.23	64.9	0.090
**Aβ-40**	126	92.1–160.0	140	104.5–164.5	11.1	0.151
**Aβ-42**	5.09	2.47–6.55	5.19	3.80–7.01	1.96	0.046*

The quantitative values of DANA neurocognitive tests (simple reaction time, procedural reaction time (PRT), and Go—No Go time, milliseconds) and serum biomarkers (GFAP, UCH-L1, Nf-L, tau, Aβ-40, and Aβ-42 [pg/mL] derived from subjects tested before (pre-) and after (post-) overpressure exposure are indicated. Values are displayed as the median and IQR.

**Fig 1:**
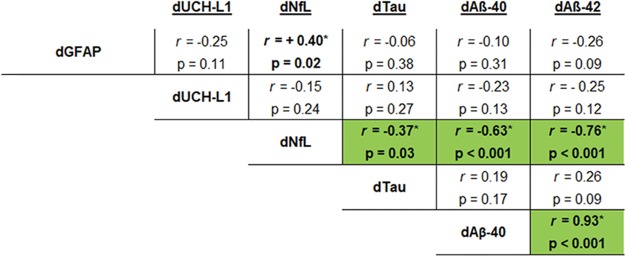
Correlation between biomarker changes. The mathematically derived changes (delta (d) = post-pre) of biomarker levels were compared to one another using Spearman rank correlation analysis after outlier removal (ROUT = 1%). Values that were statistically significant are indicated (green highlight, *p ≤ 0.05, 1-tailed Spearman rank correlation).

Changes in biomarker levels derived from the delta (d = post–pre) were evaluated among of individual study participants using 1-tailed Spearman rank correlations after outlier removal (Fig 1). Delta Nf-L had a positive relationship with dGFAP (r = +0.40, p = 0.020). Interestingly, suppression of dNf-L was associated with higher dAβ-40 (r = -0.63, p < 0.001), dAβ-42 (r = -0.76, p < 0.001), and dTau (r = -0.37, p = 0.028); thus, each comparison has a negative correlation. As expected, dAβ-40 had a high degree of concordance with dAβ-42 (r = +0.93, p < 0.001). Overall, evaluation of inter-subject changes show that suppressed GFAP and NfL are generally associated with increased levels of tau, Aβ-40, and Aβ -42 as a consequence of Exp. The remaining comparisons were not significant.

Next, relationships between the top three symptoms (headaches, dizziness, and taking longer to think), the changes in SRT, and that of biomarkers that showed effects after Exp were determined. Correlation analysis indicated that there was no relationship between dSRT, dPRT, or dGNG compared to the changes in biomarker levels (Table 4).

**Table 4:** Relationship between changes in DANA and biomarker levels after overpressure exposure.

	dSRT		dPRT		dGNG	
	Spearman *r*	p-value	Spearman *r*	p-value	Spearman *r*	p-value
**dGFAP**	0.15	0.215	-0.09	0.329	-0.41	0.013
**dUCH-L1**	-0.17	0.196	0.06	0.382	0.14	0.248
**dNfL**	-0.01	0.480	-0.10	0.311	-0.27	0.087
**dTau**	-0.08	0.347	-0.27	0.078	-0.08	0.344
**dAb40**	0.20	0.152	-0.05	0.404	-0.12	0.272
**dAB42**	0.15	0.222	-0.02	0.461	0.03	0.448

The change (delta (d) = post pre) in SRT was compared to changes in biomarker levels as displayed. The biomarker, correlation coefficient, and p-value are indicated for each comparison. Statistically significant comparisons are indicated (* p ≤ 0.05, 1-tailed Spearman correlation).

The top three symptoms (headaches, dizziness, and/ or taking longer to think) as well as mTBI/concussion history based on self-reporting, were dichotomized into two groups exemplifying participants who reported either a decrease or no change (—) compared to those who reported an increase (+) in symptomology or prior mTBI/concussion (S3 Table). This dichotomized data was compared to changes in DANA metrics and biomarker levels after outlier removal.

**Fig 2:**
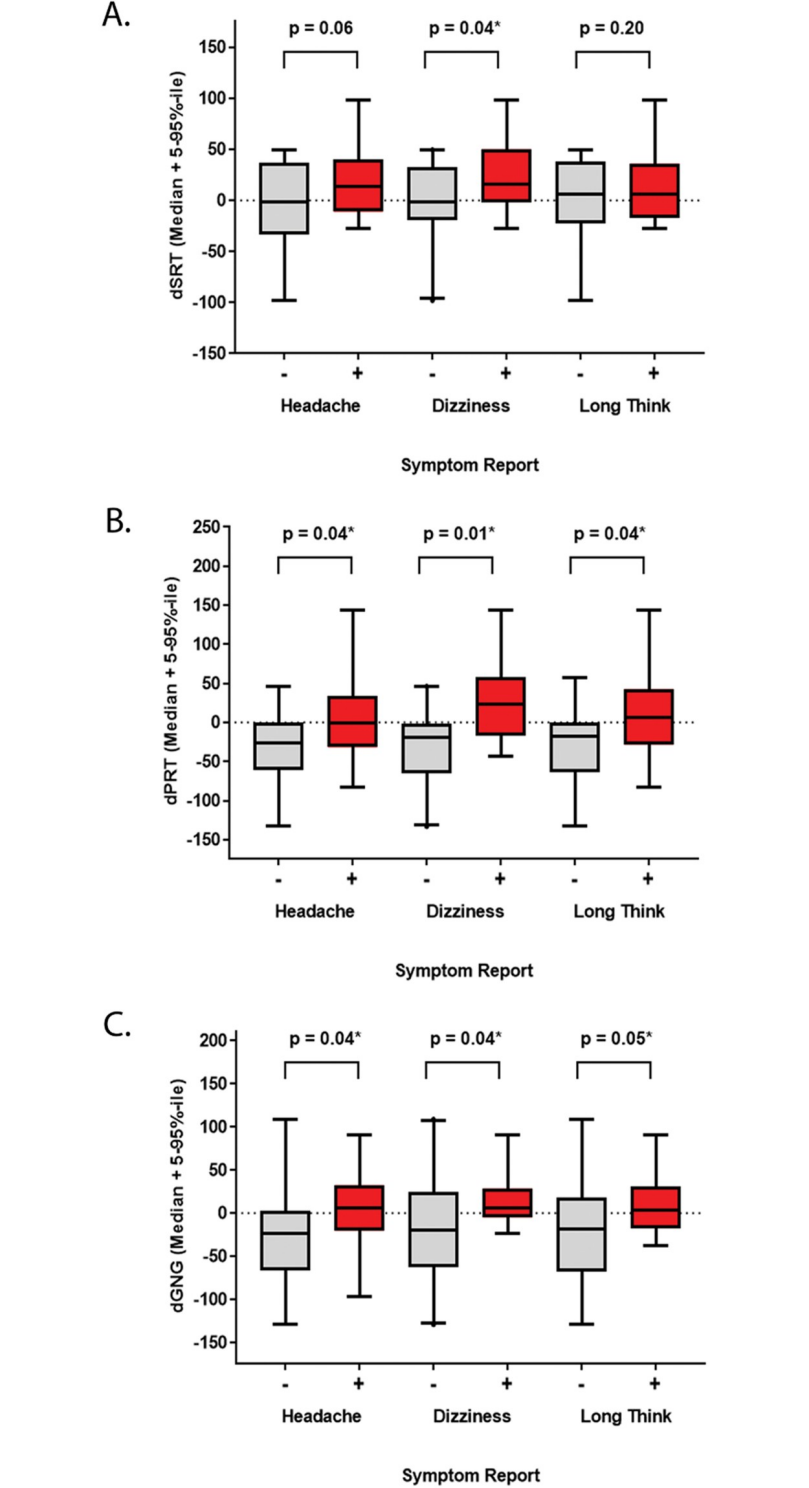
Relationships between changes in neurocognitive tests and symptoms. The change (delta (d) = post pre) in DANA metrics were compared to symptomology standardized as binary values. Data is displayed as a box plot (median + 5–95%-ile range) for the content of each DANA metrics (x-axis) among participants who reported no change or a decrease (grey, -) compared to an increase (red, +) in headaches, dizziness or taking longer to think (y-axis). (A) dSRT, (B) dPRT, (C) dGNG. Comparisons were conducted after outlier removal (ROUT = 1%). Statistically significant comparisons are indicated (* p ≤ 0.05, unpaired 1-tailed Mann-Whitney or Welch’s t-Test as appropriate).

As expected, DANA metrics were generally associated with symptomology (Fig 2). Delta SRT was slower (exemplified by an increased value) in participants who reported dizziness (decreased or no change—median: -1.38, range: -97.9 to 49.4, n = 20; increased–median: 16.2, range: -27.3 to 98.6, n = 9, p = 0.04) (Fig 2A). Values were also greater among those who reported headaches (decreased or no change—median: -1.38, range: -97.9 to 49.4, n = 14; increased–median: 14.0, range: -27.3 to 98.6, n = 15; p = 0.06) or taking longer to think (decreased or no change—median: -5.85, range: -97.9 to 49.4, n = 17; increased–median: 6.44, range: -27.3 to 98.6, n = 12, p = 0.20), but these comparisons were not significant. Although, median distribution of PRT and GNG were not significant using pre- and post-Exp comparisons, higher dPRT levels were associated with headaches (decreased or no change—median: -26.1, range: -133 to 46.0, n = 14; increased–median: -0.38, range: -82.8 to 144, n = 15, p = 0.04), dizziness (decreased or no change—median: -18.6, range: -133 to 46.0, n = 20; increased–median: 22.8, range: -42.7 to 144, n = 9, p = 0.01) and taking longer to think (decreased or no change—median: -18.4 range: -133 to 57.2, n = 17; increased–median: 5.74, range: -82.8 to 144, n = 12, p = 0.04) (Fig 2B). A similar profile for symptoms in relation to dGNG, such that comparisons to headaches (decreased or no change—median: -23.6, range: -129 to 109, n = 14; increased–median: 6.33, range: -96.6 to 91.1, n = 15, p = 0.04), dizziness (decreased or no change—median: -19.4, range: -129 to 109, n = 20; increased–median: 6.33, range: -23.0 to 90.9, n = 9, p = 0.04), and taking longer to think (decreased or no change—median: -18.1, range: -129 to 109, n = 17; increased–median: 3.87, range: -37.1 to 90.9, n = 12, p = 0.05) were also significant (Fig 2C).

**Fig 3:**
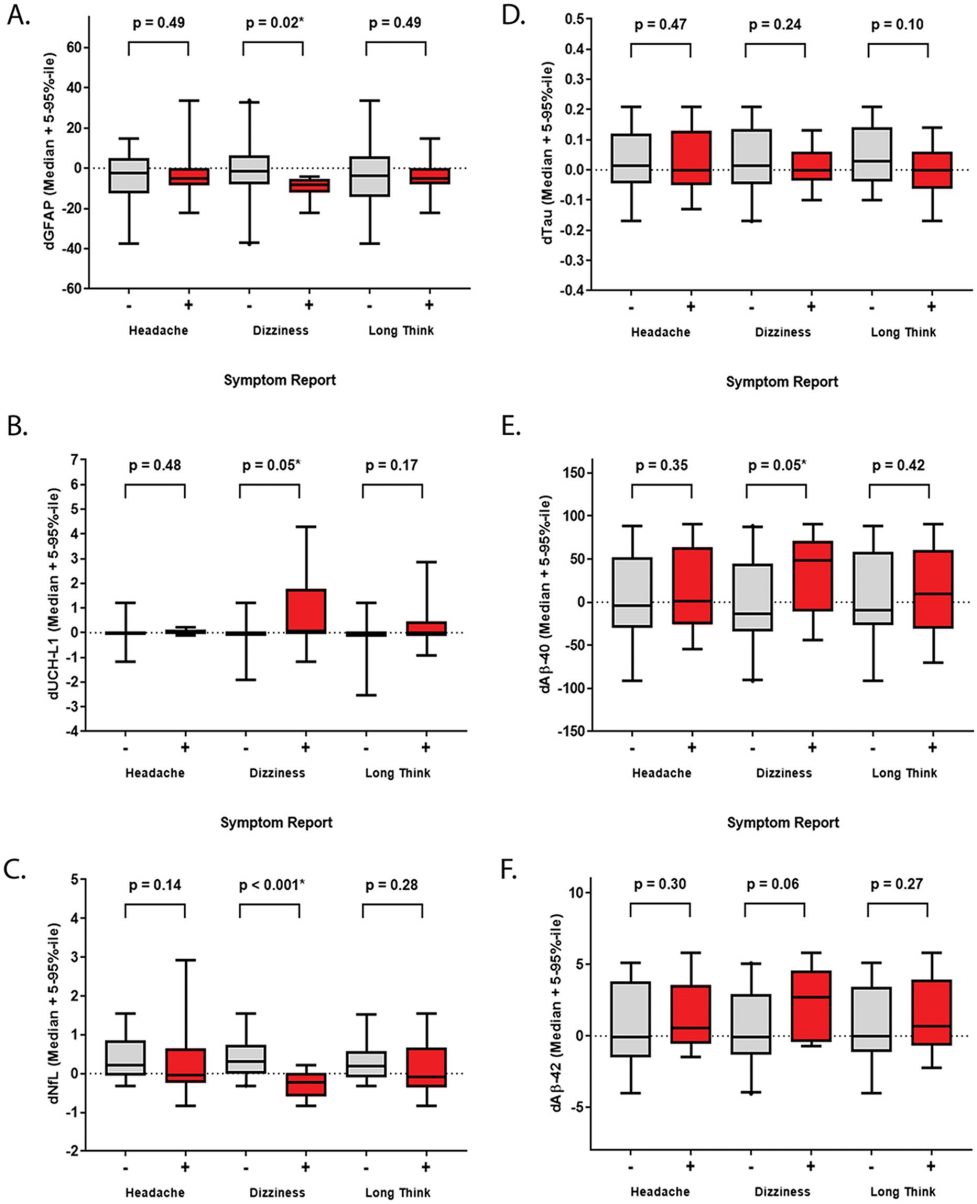
Relationships between changes in biomarker levels and symptoms. The change (delta (d) = post pre) in biomarker levels were compared to symptomology standardized as binary values. Data is displayed as a box plot (median + 5–95%-ile range) for the content of each biomarker (x-axis) among participants who reported no change or a decrease (grey, -) compared to an increase (red, +) in headaches, dizziness or taking longer to think (y-axis). (A) dGFAP, (B) dUCH-L1, (C) dNf-L, (D) tau, (E) dAβ-40 (F) dAβ-42. Comparisons were conducted after outlier removal (ROUT = 1%). Statistically significant comparisons are indicated (* p ≤ 0.05, unpaired 1-tailed Mann-Whitney or Welch’s t-Test as appropriate).

Next, delta biomarker values were compare to symptomology and indicated that changes in biomarker levels were not linked to headaches or taking longer to think experience after Exp (Fig 3). In contrast, the decreased value of dGFAP was associated with dizziness (decreased or no change—median: -1.57, range: -37.7 to 33.6, n = 20; increased–median: -8.25, range: —22.0 to -4.00, n = 8, p = 0.02) (Fig 3A), whereas dTau was not related to any symptoms (Fig 3B). Higher dUCH-L1 (decreased or no change—median: 0, range: -1.90 to 1.20, n = 17; increased–median: 0, range: -1.16 to 4.27, n = 9, p = 0.05) (Fig 3C). However, this result may be skewed by two study participants who had high dUCH-L1 levels even after outlier removal (S2 Table). Suppressed dNfL (decreased or no change—median: 0.30, range: -0.31 to 1.55, n = 19; increased–median: 0.24, range: -0.83 to 0.22, n = 8, p = 0.05) (Fig 3D) and elevated dAβ-40 (decreased or no change—median: -13.5, range: -91.1 to 88.2, n = 20; increased–median: 48.2, range: -44.4 to 90.3, n = 9, p < 0.001) (Fig 3E) values were also associated with post-Exp dizziness. Delta Aβ-42 held a similar trend (decreased or no change—median: -0.09, range: -4.04 to 5.08, n = 20; increased–median: 2.73, range: -0.72 to 8.81, n = 9, p = 0.06), but was not significant (Fig 3F). Changes in DANA metrics were, as expected, aligned with the top three symptoms reported. Interestingly, biomarker levels, particularly GFAP or NfL suppression and AB elevation, occurred in participants who reported post-Exp dizziness.

DANA and biomarker changes were compared to dichotomized mTBI/concussion history reporting. Although the range among participants who reported former mTBI/concussion was greater, median values of dUCH-L1 were equivalent (No–median: 0.00, range: -2.54 to 1.20, n = 19, Yes—median: 0.00, range: -0.18 to 4.27, n = 9, p = 0.05) (S3 Table). As with comparisons to symptomology, these results are skewed by a few participants. No other trends were observed.

## Discussion

Quantitation of peripheral biomarkers offers a means to objectively monitor the effects of overpressure Exp within groups and among individuals involved in military training operations. Measurement of acute biomarkers remains sparse, particularly if the individual does not have outwardly obvious, clinically defined mTBI or concussion marked by well-known symptoms, such as loss of consciousness or changes in gait. Therefore, this study compared serum biomarker levels, neurocognitive deficits, and reported symptoms caused before and within one hour after mild-moderate Exp among military breachers within a single training session. The main findings show that median elevation of Nf-L, tau, Aβ-40 or -42, but a suppression of GFAP was evident in serum collected one hour post-Exp compared to pre-Exp sampling. Changes in DANA metrics were aligned with the top three symptoms reported and serum GFAP, NfL, and Aβ peptide changes were largely associated post-Exp dizziness.

### A subset of TBI-related proteins are potential biomarkers of acute overpressure exposure based on evaluation of group effects

The utility of blood-based biomarkers are increasingly investigated for concussion or subconcussion. Breacher’s brain symptomology caused by overpressure exposure is similar to that of mTBI or concussion. Therefore, we hypothesized that biomarkers would also have utility for symptomatic overpressure exposure.

GFAP and UCH-L1 are, perhaps, the most thoroughly studied as biomarkers for moderate-severe TBI or closed head hemorrhagic injury. Levels dramatically increase in the serum or plasma within 12-24h [13–16]. GFAP and UCH-L1 are not typically evident in patients with an mTBI unless hemorrhage or intracranial lesions are presented [17, 18]. However, composite assessment of GFAP and UCH-L1 (in addition to spectrin break down product (SBDP-150) revealed that these proteins were elevated in blood collected from study participants who had the most striking decrements in neurocognitive performance, including simple reaction time, as well as symptom reporting [19] in the absence of a clinically defined concussion. UCH-L1 was also increased in serum collected two weeks after training from a subset of participants, but these results were not linked to neurocognitive performance or symptomology [20]. Participants in the present study do not suffer from complicated mTBI within this context. The lack of a robust post-exposure UCH-L1 response among the cohort is not surprising. The moderate drop in median GFAP levels is not known in the context of physiology. However, this observation presents a novel observation that may be deserving of further investigation, specifically in the context of overpressure exposure and its systemic outcomes.

Nf-L and tau are two of the most abundant cytoskeletal proteins in both the peripheral (PNS) and central nervous system (CNS). Both have recently become more prevalent as potential biomarkers of brain trauma, neurological disease, and repeated concussion [21–23]. In the context of low level exposure without a direct impact to the head, this study indicated that median levels of serum Nf-L and tau were elevated (although tau did not meet statistical thresholds). Exposure is reported to impact brain tissues in a way that may mirror a sub-concussive event [24, 25], causing cytoskeletal abnormalities and demyelination in rodent models [26–28]. In animal models, serum tau is elevated within 6h-1d of and the heavy chain isoform of neurofilament is increased within 2h after mild exposure [29]. Serum Nf-L is reported to increase acutely, after sub-concussive head impacts when viewed in the context of TBI status [30]. Both proteins are elevated in blood 1-6h hour after play among athletes who have prolonged return to play status [31–33].

This work indicated that median Aβ-42 was elevated one hour post-exposure. Aβ peptides are toxic monomers shown to be crucial to pathogenesis of chronic neurodegenerative diseases, such as Alzheimer’s disease (AD) [34] or chronic traumatic encephalopathy [35]. Aβ is increased in the brains derived from veterans with a history of between chronic exposures caused by blast, which may offer associative or causative relationship to neurodegenerative diseases. Assessment of Aβ levels in blood is primary viewed in the context of cognitive decline or advanced age wherein Aβ levels in the blood typically decrease in accordance with increased plaque burden in the brain [36]. The effect of Aβ in blood collected from cognitively normal, yet acutely injured and symptomatic, subjects is not fully understood. Recently, increased levels Aβ peptides have been detected in the blood of active duty and veteran populations. Plasma Aβ-40 is elevated among service members who sustained a clinically diagnosed TBI or experienced chronic symptoms associated with PTSD [37, 38]. Interestingly, increased serum Aβ is also associated with hypoxia or hypoxemia [39, 40], which is proposed to occur as a consequence of altered cerebral blood flow after blast overpressure exposure [41, 42].

When study participants are viewed collectively, assessment of median biomarker values indicate that serum GFAP and NfL decrease while Aβ-42 increases after overpressure exposure. These biomarker changes occur within the same time frame as SRT decrement, although there is no overall correlation to the DANA metrics within the cohort of participants. Dichotomizing biomarker changes according to breacher’s brain symptomology may offer additional insight regarding individual and group post-exposure responses.

### Acute shifts in biomarker levels and neurocognitive decrements are associated with post-exposure symptomology, not mTBI or concussion history

Breacher’s brain symptomology, such as post-exposure headaches, dizziness and taking longer to think, are well established [43], yet typically studied in relation to mTBI/concussion diagnosis and chronic blast exposure. The current study indicated that low levels of overpressure exposure consistently have this effect, even at early time frames. Neurocognitive decrements have been proven to be useful during acute timeframes [44]. Therefore, association of SRT, PRT, and GNG decrements with the top three reported symptoms among participants is fitting for blast exposure.

Interestingly, decreased GFAP or NfL, elevated UCH-L1, and, to a greater extent, increased Aβ peptide levels were detected in serum of participants who reported increased dizziness after exposure. Acute GFAP and NfL suppression among symptomatic participants was surprising, and may appear to be contrary to the effects shown for concussed athletes, wherein biomarkers generally increase among cohorts with a clinically defined concussion [32]. However, it is notable the pre-game levels were not determined and that the temporal dynamics indicate a decrease 1–12 hours after play. Suppressed GFAP in relation to symptomology remains a novel observation.

The change in serum UCH-L1 was positive (e.g. increased) among the subset of participants who reported post-exposure dizziness. Previously, UCH-L1 levels were shown to be unchanged in serum derived from symptomatic concussion patients who were negative for CT abnormalities [45]. However, UCH-L1 was elevated in serum one hour after game-play among subconcussive football players, although the relationship to specific symptoms were not determined [46].

Direct relevance of acute Aβ-peptide elevation is not well known in the context of symptomology caused by overpressure exposure or subconcussive paradigms. Rather, fluctuation in blood Aβ are largely understood in the context of subacute-chronic symptomatic mTBI, after activities such as boxing, without stratification of specific symptoms [47]. However, mass spectrometry-based proteomics of serum revealed peptides (proteins) that were specifically associated with the decree of PTSD or post-concussive syndrome symptomology among veterans who suffered a mTBI [48]. It is possible that acute Aβ elevation among symptomatic participants in this study are aligned with these observations. Overall, this study is the first to show dysregulation of blood biomarkers, specifically Aβ, are aligned with symptomology that is common among participants exposed to blast overpressure.

This study is not without a few potential caveats. First, symptomology and mTBI/concussion history may be under-reported [49–51]. Changes in UCH-L1 levels were higher in serum of participants who reported prior mTBI/concussion based on self-report. However, definitive medical evidence is not available. The change in UCH-L1 after low levels of overpressure exposure is considered a small effect that will remain under consideration for future studies. Second, amyloid precursor protein expression and release of Aβ peptides may occur outside of the CNS, including the epidermis and muscle and leak into the blood stream [52, 53]. However, there were no reports of tissue injury among participants. Lastly, circadian variation of Aβ levels among healthy controls (< 5%) [54, 55] has been reported, but the changes induced by exposure within this study eclipsed those found associated with circadian patterns.

To our knowledge, this work is the first to explicitly report changes in biomarker levels in the context of acute overpressure exposure compared to pre-exposure values. The strict definition of a clinical mTBI/concussion was not met within this paradigm. However, acute measurement of proteins in serum, particularly Aβ peptides, coupled with symptomology and neurocognitive assessment may provide a novel biomarker relevant to subconcussive effects of blast overpressure exposure. Acute serum biomarker dynamics among overpressure-exposed persons, who have subconcussive breacher’s brain symptoms, may be worth further collective evaluation particularly in the absence of a clinically defined mTBI/concussion.

## Conclusions

Mild-moderate blast exposure is associated with acute elevation of serum Aβ peptides, a slight increase in tau, but a reduction in GFAP and NfL. Reaction time decrements and these biomarker profiles were collectively associated with post-exposure dizziness. Acute evaluation of serum protein levels, well-known neurocognitive tests, and symptoms before and after exposure have the potential to serve as a multiplexed surrogate biomarkers of exposure in absence of a direct impact to the head. These metrics may be adaptable to field-ready tools and aid return to duty decisions independent of TBI status.

## Supporting information

S1 Table Field metrics, biomarker levels, and self-reports of study participants before and after overpressure exposure. The age (years), peak pressure (psi, kPa), impulse (psi-ms), DANA metrics (milliseconds), quantitative biomarker values [pg/mL], changes in symptomology (0 = Decrease or No Change), and mTBI/concussion history (0 = No, 1 = Yes) are shown for each study participant prior to outlier removal or statistical analysis. Missing data for responses is indicated as no data (ND).
(XLSX)

S2 Table Self-reported symptomology of participants. Self-reported symptoms from the 32-item survey for each study participant are shown. (A) Pre-exposure and (B) Post-exposure data is displayed in the Likert scale format from none (0) to severe (4).
(XLSX)

S3 Table Relationship between changes in DANA or biomarker levels with symptomology or mTBI/concussion history based on self-reporting. Dichotomized symptoms (headaches, dizziness, and taking longer to think) or mTBI/concussion reporting is shown in relation to changes (post–pre delta) in DANA metrics or biomarker levels. (A) dSRT, (B) dPRT, (C) dGNG, (D) dGFAP, (E) dUCH-L1, (F) dNf-L, (G) tau, (H) dAβ-40 (I) dAβ-42. Data is shown after outlier removal (ROUT = 1%) and p-values are indicated (* p ≤ 0.05, unpaired 1-tailed Mann-Whitney or Welch’s t-Test as appropriate).
(XLSX)
